# Evaluating Asthma Mobile Apps to Improve Asthma Self-Management: User Ratings and Sentiment Analysis of Publicly Available Apps

**DOI:** 10.2196/15076

**Published:** 2020-10-29

**Authors:** Marlene Camacho-Rivera, Huy Vo, Xueqi Huang, Julia Lau, Adeola Lawal, Akira Kawaguchi

**Affiliations:** 1 Department of Community Health Sciences SUNY Downstate Health Sciences University Brooklyn, NY United States; 2 Department of Computer Science Grove School of Engineering City College of New York New York, NY United States; 3 Department of Community Health and Social Medicine CUNY School of Medicine New York, NY United States

**Keywords:** mHealth, asthma apps, sentiment analysis, user ratings, smartphone, mobile phone

## Abstract

**Background:**

The development and use of mobile health (mHealth) apps for asthma management have risen dramatically over the past two decades. Asthma apps vary widely in their content and features; however, prior research has rarely examined preferences of users of publicly available apps.

**Objective:**

The goals of this study were to provide a descriptive overview of asthma mobile apps that are publicly available and to assess the usability of asthma apps currently available on the market to identify content and features of apps associated with positive and negative user ratings.

**Methods:**

Reviews were collected on June 23, 2020, and included publicly posted reviews until June 21, 2020. To characterize features associated with high or low app ratings, we first dichotomized the average user rating of the asthma app into 2 categories: a high average rating and a low average rating. Asthma apps with average ratings of 4 and above were categorized as having a high average rating. Asthma apps with average ratings of less than 4 were categorized as having a low average rating. For the sentiment analysis, we modeled both 2-word (bi-gram) and 3-word (tri-gram) phrases which commonly appeared across highly rated and lowly rated apps.

**Results:**

Of the 10 apps that met the inclusion criteria, a total of 373 reviews were examined across all apps. Among apps reviewed, 53.4% (199/373) received high ratings (average ratings of 4 or 5) and 47.2% (176/373) received low ratings (average ratings of 3 or less). The number of ratings across all apps ranged from 188 (AsthmaMD) to 10 (My Asthma App); 30% (3/10) of apps were available on both Android and iOS. From the sentiment analysis, key features of asthma management that were common among highly rated apps included the tracking of peak flow readings (n=48), asthma symptom monitoring (n=11), and action plans (n=10). Key features related to functionality that were common among highly rated apps included ease of use (n=5). Users most commonly reported loss of data (n=14) and crashing of app (n=12) as functionality issues among poorly rated asthma apps.

**Conclusions:**

Our study results demonstrate that asthma app quality, maintenance, and updates vary widely across apps and platforms. These findings may call into question the long-term engagement with asthma apps, a crucial factor for determining their potential to improve asthma self-management and asthma clinical outcomes.

## Introduction

### Background

Asthma is the leading chronic disease in children and adolescents and one of the most common among adults, with more than 25 million Americans impacted [1]. Nationally, asthma accounts for 11 million doctor’s office visits, 439,435 discharges from hospital inpatient care, and 1.7 million emergency department visits each year [2]. Further, economic costs associated with asthma are significant, both for the United States and for asthmatics and their families. From 2008 to 2013, the annual economic cost of asthma was more than US $81.9 billion, including medical costs and loss of work and school days: US $3 billion in losses due to missed work and school days, US $29 billion due to asthma-related mortality, and US $50.3 billion in medical costs [2].

Many factors combine to contribute to poor asthma rates and worsen outcomes. Provider practice behaviors, suboptimal access to health care, lack of patient knowledge regarding proper medication use, and patients’ difficulty adhering to medical regimens contribute to poor asthma outcomes [3-8]. With appropriate medical care including education, patients can achieve asthma control; however, there are competing challenges to providing effective patient education, such as time constraints and prioritizing other issues above asthma education [9].

Mobile health apps and devices to monitor and track health-related questions are a rapidly growing field within the public health, data science, and technology sectors [10-16], and offer potential opportunities to address some of the barriers to patient asthma education [17-20].

However, promotion of new technologies is predicated on the hypothesis that enabling people to quantify their own behaviors will drive health behavior change through contextualization and goal setting [21]. Mobile apps (configured to work independently or with wearable devices) can be used to record and track health-related behaviors, provide tailored education, and send reminders and prompts. Nationally, approximately 80% of US adults own a smartphone and close to 60% of smartphone owners also report installing one or more health apps onto their smartphones [22,23].

In recent years, there has been a proliferation of new mobile apps for the self-assessment and self-management of asthma [24,25]. However, uptake of asthma apps has been sparse, despite evidence of their efficacy in impacting asthma outcomes [26]. Results from randomized controlled studies show that use of mobile health apps improves asthma symptoms and medication compliance, which should, in turn, reduce emergency department visits and hospital admissions [27-29]. To our knowledge, there has not been a comprehensive review of asthma apps which considers user ratings, features, and reviews as a tool to identify potential barriers and facilitators of app use among individuals self-managing asthma.

### Objectives

The aims of this study were (1) to provide a descriptive overview of asthma mobile apps that are publicly available and (2) to assess the usability of asthma apps currently available on the market to identify content and features of apps associated with positive and negative user ratings.

## Methods

### Asthma App Selection

To examine the average user rating and reviews for asthma apps, the following inclusion criteria were applied for our search: apps must be available for download on Android or iOS platforms, written in English, available within the United States, able to be downloaded onto a smartphone or tablet, and had at least one update within the last 5 years. Apps must also have a primary focus on asthma, with respect to either asthma education or asthma self-management. Because of the potential for bias in ratings due to small sample size, only apps that had more than 10 written reviews were included. Apps were not excluded based on cost for use or intended audience. We started with a comprehensive list of apps that strictly matched our inclusion criteria. The list was expanded by using the following keywords commonly associated with asthma education or asthma management to identify additional asthma apps: *asthma management*, *asthma game*, *asthma quiz*, *peak flow*, and *asthma education*. All apps were evaluated by the research team based on inclusion criteria; of the 51 apps initially reviewed, 29 apps had been updated in the last 5 years. Of the 29 apps recently updated, 19 were excluded based on having less than 10 written reviews available. The final list of 10 apps included in the analysis is presented in Table 1.

**Table 1:** List of asthma mobile apps included in analysis.

Name	Developer	Android	iOS	Last update	Update times	Category
My Asthma App	Asthma and Respiratory Foundation NZ	N/A^a^	X	May 5, 2019	6	Education
Propeller	Reciprocal Labs	X	N/A	March 28, 2019	25	Management
SaniQ Asthma	Qurasoft GmbH	X	X	March 19, 2019	25	Management
Asthma Tracker	Kantonsspital Baselland	N/A	X	March 13, 2019	12	Management
AirCasting	HabitatMap	N/A	X	March 7, 2019	N/A	Environmental Data
Peak Flow	Ben Hills	X	N/A	April 26, 2018	N/A	Management
My Asthma Pal	Children’s Medical Center of Dallas	X	X	March 7, 2019	6	Management
asthmaTrack	dangerDown LLC	N/A	X	January 18, 2018	28	Management
Breathcount asthma control	Segfoltas	X	N/A	January 9, 2017	N/A	Management
AsthmaMD	AsthmaMD, Inc.	X	X	March 10, 2017	21	Management

^a^N/A: Not available

### User Ratings

To identify specific sentiments within language characteristics of user reviews that are associated with high or low app ratings, we first dichotomized the average user rating of the asthma app into 2 categories: a high average rating and a low average rating. Asthma apps with average ratings of 4 and above were categorized as having a high average rating. Asthma apps with average ratings of less than 4 were categorized as having a low average rating.

### Sentiment Analysis of User Reviews: N-Gram models

An *N-gram* is a contiguous sequence of n items from a piece of article, sentence, or speech. An *N-gram model* is a probabilistic language model for predicting the next item given the sequence of *(n–1)–gram,* which simulates characteristics of the language used in a certain corpus. Its simplicity and scalability enable its application in sentiment analysis of the reviews [30]. For this analysis, we modeled both 2-word (bi-gram) and 3-word (tri-gram) phrases that commonly appeared across highly rated and lowly rated reviews. Because of the limited number of reviews, the tri-gram model did not yield expressive results, as it identified the same tri-gram *peak flow meter* in both the good and bad reviews. The bi-grams extracted from the model showed distinct patterns in good reviews and bad reviews. As such, results from bi-gram models for highly rated reviews and lowly rated reviews are discussed in the next section. We removed the stop words in the preprocessing step, and separated the reviews into good reviews (ratings >3) and bad reviews (ratings ≤3). Reviews were collected on June 23, 2020, and contain reviews from January 08, 2010, to June 21, 2020. Of the 10 apps that met the inclusion criteria, a total of 373 reviews were examined across all apps.

## Results

### Asthma App Descriptive Results

Of the 10 apps included in the analysis, 6 were available at no cost to users and 4 apps were available for purchase, with a maximum cost of US $2.99; 50% of apps (n=5) were updated in the past 2 years.

**Figure 1:**
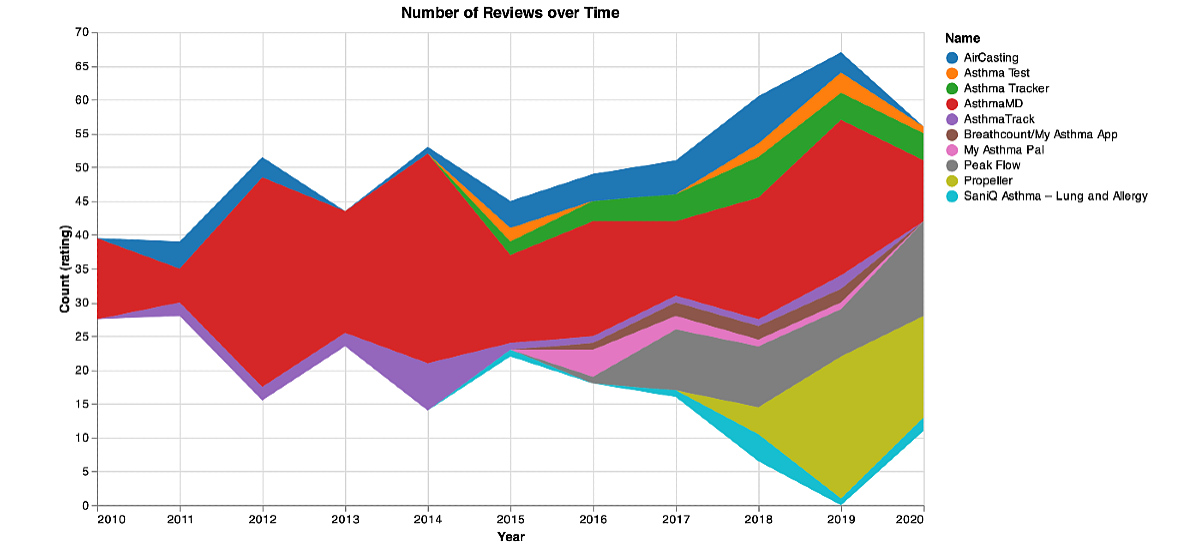
Stream graph of the review counts for asthma apps from 2010 to 2020.

**Figure 2:**
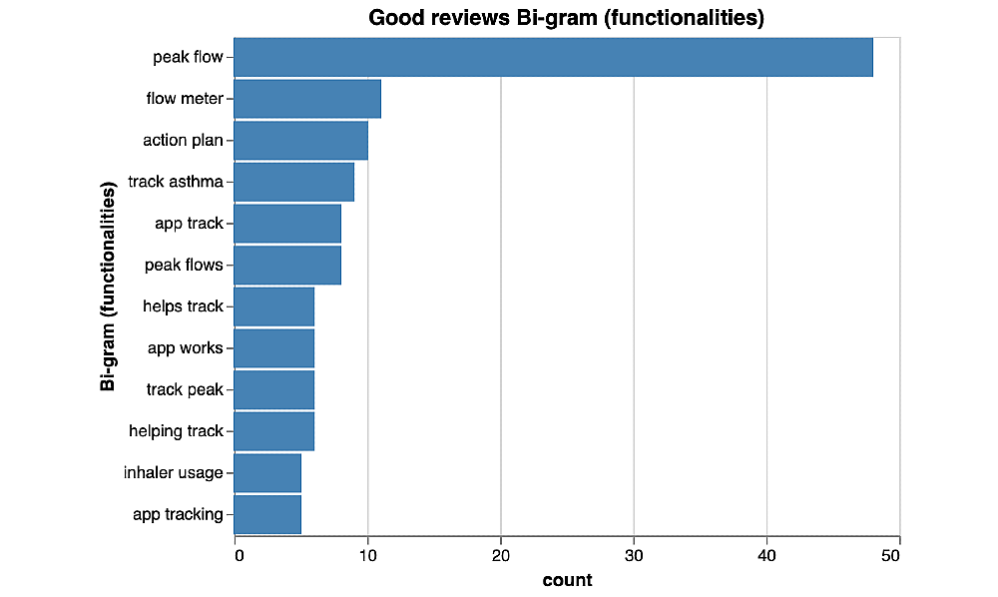
Bi-gram results of functionalities of highly rated asthma mobile apps.

**Figure 3:**
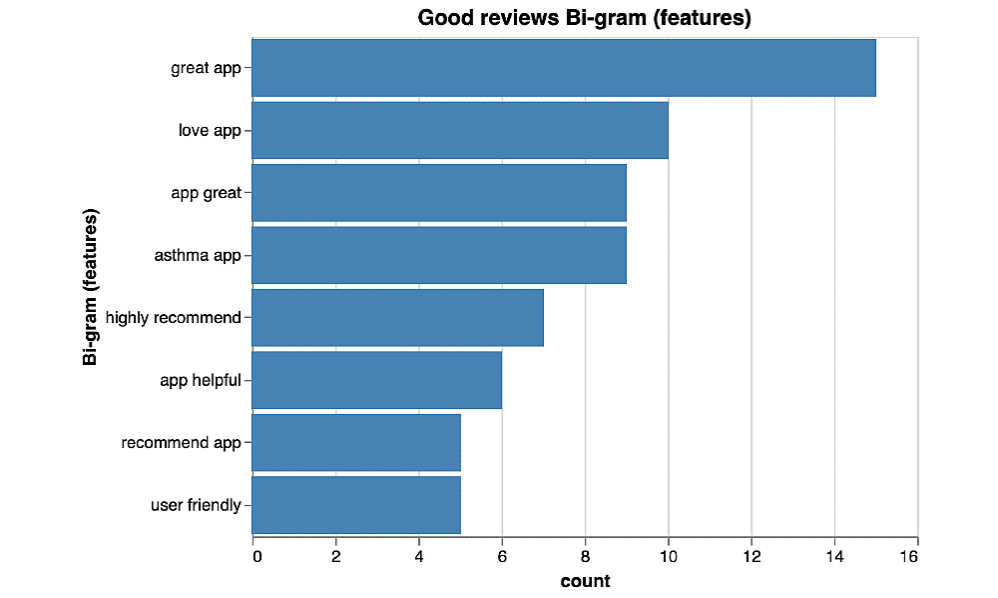
Bi-gram results of features of highly rated asthma mobile apps.

As shown in Figures 1-3, among apps reviewed, 53.4% (199/373) received high ratings (average ratings of 4 or 5) and 47.2% (176/373) received low ratings (average ratings of ≤3). The number of ratings across all apps ranged from 188 (AsthmaMD) to 10 (My Asthma App). Three of the apps were available on both Android and iOS platforms.

Stream graph of the number of review counts from 2010 to 2020 is shown in Figure 1. The number of reviews for each app is displayed in a distinct color to visually track the change in number of reviews over time. The height of the colored area suggests the number of reviews an app gets in the year. The width of the colored area suggests the total number of reviews an app gets over the years. As shown in Figure 1, the dominant app is AsthmaMD, which reached a peak number of reviews during 2012 to 2014.

### Sentiment Analysis of User Reviews

Distinct patterns of language were observed when comparing highly rated and poorly rated apps. Among the highly rated reviews, bi-grams emerged with respect to features as well as functionality of the app. Figures 2 and 3 depict the frequencies of the most commonly occurring bi-grams among highly rated apps. Several key features of asthma management that were common among highly rated apps included the tracking of peak flow readings (n=48), asthma symptom monitoring (n=11), and action plans (n=10). Key features related to functionality that were common among highly rated apps included ease of use (n=5), as illustrated in Figure 2.

**Figure 4:**
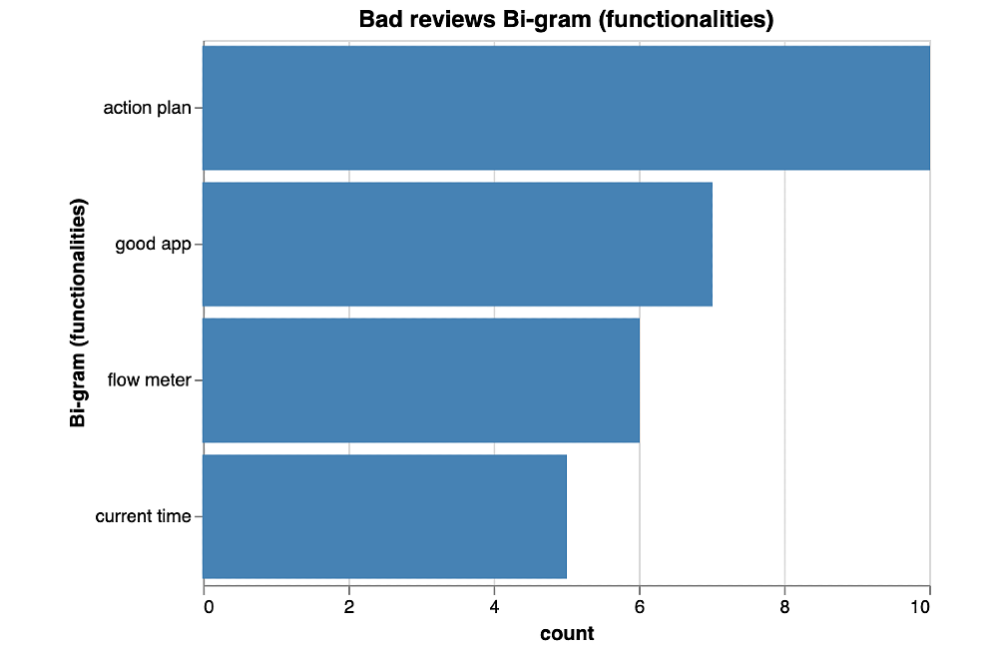
Bi-gram results of functionalities of poorly rated asthma mobile apps.

**Figure 5:**
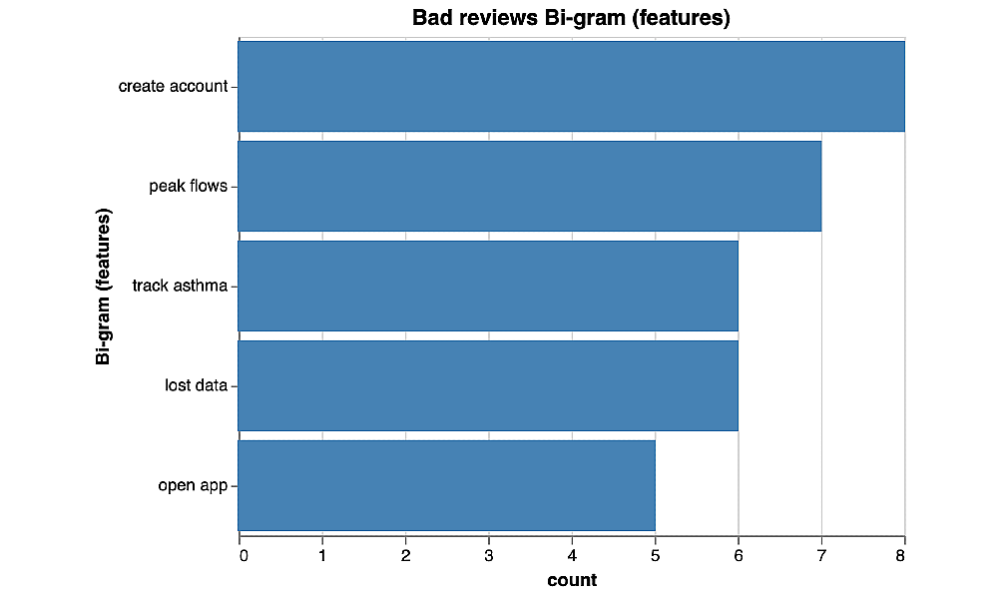
Bi-gram results of features of poorly rated asthma apps.

**Figure 6:**
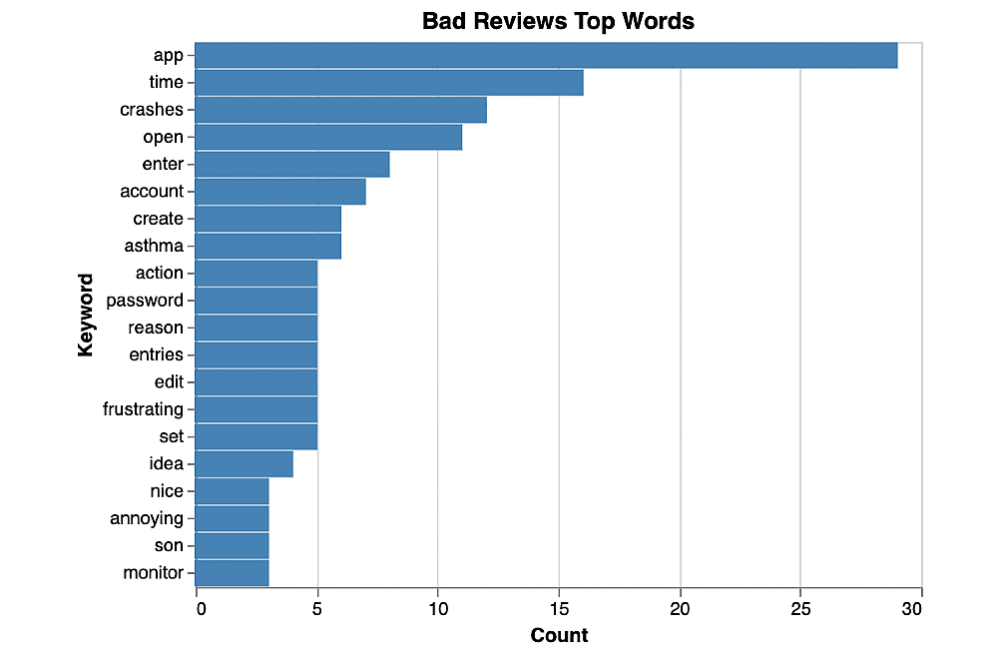
Top words in reviews of poorly rated asthma apps.

Among poorly rated asthma apps, bi-grams (Figures 4 and 5) predominantly focused on functionality issues encountered by users (Figure 4). The functionality keywords that had the most common occurrences in bad reviews were create account (n=8), lost data (n=6), open app (n=5). By identifying the most important words (or keywords) in negative reviews through the term frequency–inverse document frequency measure in Figure 6, we concluded that users most commonly reported loss of data (n=14) and crashing of app (n=12) as functionality issues among poorly rated asthma apps.

## Discussion

### Principal Results

This study analyzed ratings and features of publicly available asthma apps to identify user preferences. Our descriptive results confirmed those of prior studies which observed the limited availability of publicly available, up-to-date asthma apps on the market [26]. In terms of available functions, we observed that most asthma apps offered functions associated with several recommendations of effective self-management including asthma education, peak flow monitoring, and an action plan [31]. Consistent with prior reviews of asthma apps, our results confirmed that asthma apps offered either education or management, with few offering a combination of both [25].

Furthermore, our study results demonstrated that asthma app quality varied widely, ranging anywhere from an average user rating of 1.5 out of 5 to 5 out of 5. It also appeared that individual asthma apps varied in their user rating between Android and iOS platforms. These findings may call into question the long-term engagement with asthma apps, a crucial factor for determining their value [32,33]. In other domains such as diabetes self-management, researchers have observed that long-term engagement of app users is generally limited [34]. However, chronic diseases such as asthma require long-term self-management.

### Recommendations for App Features and Functions

With the *N-grams* model, we were able to capture several characteristics related to features and functionality that users like or dislike about the apps; for example, *peak flow* and *action plan* were frequently mentioned among features, whereas *easy to use* and *doesn’t work* were frequently mentioned around functionality. Previous studies of asthma apps confirmed peak flow tracking, symptom tracking, and medication tracking as some of the most common functions in highly rated apps [25]. Additional function recommendations which were not captured in our sentiment analysis but have been identified in previous studies of asthma apps include medical appointment tracking, health snapshots, and clinical asthma questionnaires [20,26,35]. Additional categories which have been identified through formative research of specific asthma apps include the incorporation of notification features such as those related to medication reminders, medical appointment reminders, and peak flow reading reminders [36-40].

One potential way to improve long-term engagement, which has been successfully applied to physical activity, is interactions with virtual coaches [41,42]. Thus, developers of upcoming asthma apps might consider the implementation of virtual coaches to enhance long-term engagement. Another potential method of improving long-term engagement, in particular among children and adolescents with asthma, could be through gamification and use of contingent rewards [43-45]. Lastly, application of behavior change techniques including specific goal setting, provision of performance feedback, and barrier identification may also improve acceptability and long-term engagement with asthma apps [46-48].

### Limitations

Our study did have several limitations, which should be considered when interpreting its findings. The popularity of an asthma app may not yield information regarding the quality of the asthma content presented in the app with respect to the clinical or scientific literature. Prior studies have observed variability in the quality of asthma information with respect to its consistency with National Asthma Education and Prevention Program guidelines [3]. Future studies should examine the concordance between asthma education and asthma management clinical tools within popular asthma apps.

Limitations of sentiment analysis include its sensitivity to review content; as such, user ratings may not always correspond to descriptive reviewer feedback. Further, displays of reviews were limited to downloads with a minimum threshold of 10 reviews. However, sentiment analysis has been increasingly applied across a variety of health outcomes [49-52]. Finally, this study did not include assessment of apps using a validated instrument (eg, Mobile Application Rating Scale). However, this type of analysis was conducted in a previous review of asthma apps [25].

### Conclusions

Our study extends previous research in this field by focusing on the experiences and reviews of asthmatics’ interactions with publicly available asthma apps. Our study results have several implications with respect to informing the development of asthma mobile apps and their recommendation for clinical use. As use of asthma apps have been found to have an impact on several clinical outcomes, including but not limited to control, quality of life, medication adherence, and patient-reported outcomes, improvements to asthma apps should include a focus on user-centered design and experiences. Combining big-data analytic approaches with qualitative data from users may yield additional insights to improve usability and long-term engagement with asthma apps. Further, collaborations between asthma app developers, clinicians, and researchers should include considerations regarding data security, privacy features, and sharing of personal health information which would also increase patient and provider confidence regarding recommending the use of asthma mobile apps to improve asthma self-management.

## Abbreviations

mHealth: mobile health
NAEPP: National Asthma Education and Prevention Program
